# Hemispheric Specialization in Dogs for Processing Different Acoustic Stimuli

**DOI:** 10.1371/journal.pone.0003349

**Published:** 2008-10-09

**Authors:** Marcello Siniscalchi, Angelo Quaranta, Lesley J. Rogers

**Affiliations:** 1 Department of Animal Production, University of Bari, Bari, Italy; 2 Centre for Neuroscience and Animal Behaviour, University of New England, Armidale, Australia; Victoria University of Wellington, New Zealand

## Abstract

Considerable experimental evidence shows that functional cerebral asymmetries are widespread in animals. Activity of the right cerebral hemisphere has been associated with responses to novel stimuli and the expression of intense emotions, such as aggression, escape behaviour and fear. The left hemisphere uses learned patterns and responds to familiar stimuli. Although such lateralization has been studied mainly for visual responses, there is evidence in primates that auditory perception is lateralized and that vocal communication depends on differential processing by the hemispheres. The aim of the present work was to investigate whether dogs use different hemispheres to process different acoustic stimuli by presenting them with playbacks of a thunderstorm and their species-typical vocalizations. The results revealed that dogs usually process their species-typical vocalizations using the left hemisphere and the thunderstorm sounds using the right hemisphere. Nevertheless, conspecific vocalizations are not always processed by the left hemisphere, since the right hemisphere is used for processing vocalizations when they elicit intense emotion, including fear. These findings suggest that the specialisation of the left hemisphere for intraspecific communication is more ancient that previously thought, and so is specialisation of the right hemisphere for intense emotions.

## Introduction

Behavioural and neural lateralisation is known to be widespread among non-human animals: birds, fishes, amphibians, reptiles, and mammals have been shown to display a lateralized behaviour and/or brain asymmetries [1], [2], [3], suggesting that cerebral functional asymmetry is a fundamental feature of all vertebrate brains. Activity of the right cerebral hemisphere has been associated with response to novelty and the expression of intense emotions, such as aggression, escape behavior, and fear (summarised by Rogers) [3]. Activity of the left hemisphere involves use of learned templates or rules: it categorizes stimuli and responds to features that are invariant and repeated [4].

In dogs behavioural lateralization is evident in a variety of functions, including asymmetric tail wagging and paw preferences [5], [6], [7]. Paw preference in dogs has been measured using several tasks; for example, removal of tape placed over their nose [5], removal of a blanket from over the head and retrieval food from a can [7] or from a Kong^TM^ [8]. Moreover, Branson and Rogers [8] have found that the strength of paw preference is associated with noise phobia: dogs with no significant paw preference to hold a Kong in order to obtain food from it were found to be more reactive to the sound of fireworks or a thunderstorm than were dogs with significant paw preferences.

Regarding the auditory system, both behavioural and lesioning studies suggest that the brain processes acoustic stimuli in an asymmetrical way [3]. Macaque monkeys, like humans, use the auditory system of the left hemisphere preferentially to process their species-typical vocalizations [9], [10], [11]. Hauser and Anderson [10] showed (using a head-orienting procedure) that rhesus macaques turned with the right ear leading (left hemisphere) in response to conspecific vocalizations (aggressive, fearful and affiliative calls) but turned with the left ear leading (right hemisphere) in response to a vocalization of another species (alarm call of a sea bird). In mouse lemurs, males, but not females, exhibit a significant right ear-left hemisphere bias when exposed to conspecific communication sounds [12]. A left-hemispheric advantage for the perception of species-specific vocalizations, similar to the findings for humans and primates (with the exceptions of vervet monkeys and barbary macaques) [13], [14] based on behavioral and neurological approaches, has also been described in birds (raptors, starlings) [15], [16] and non-human mammals (sea lions, mice) [17], [18]. The activity of the left hemisphere, in primates, also appears to be associated with the production of social contact calls: Hook-Costigan and Rogers [19] found that when marmosets produced social contact vocalizations, they opened the right side of the mouth wider than the left: the opposite was the case when they produced fear/mobbing calls. Hauser [20] found that, as in humans, rhesus monkeys also exhibit right hemisphere dominance for facial expressions associated with negative/withdrawal emotions, which indicates the right hemisphere's specialization for expression of fear. The same specialization of the right hemisphere for fear expression seems to apply to dogs since recent research has found that stimuli that elicit withdrawal or a fear response (e.g. seeing a dominant unfamiliar dog) are associated with higher amplitude of tail wagging movements to the left side of the dog's body, hence reflecting activation of the right hemisphere [6]. In dogs, the right hemisphere seems to be involved also in the detection of some acoustic features of the human speech: Adams and colleagues [21] recorded auditory evoked responses (AERs) from the left and right temporal and parietal scalp regions of ten 15-week-old border collies while the animals listened to series of consonant-vowel syllables in which the consonant sounds varied in voice onset time. Results showed that portions of the right-hemisphere exhibited AERs when the dogs discriminated between consonant sounds that are important for human phonetic contrast. Despite all of this evidence of lateralized processing of acoustic stimuli, so far there have been no studies on hemispheric specialization of the dog's brain in processing their own species-typical vocalizations.

In general, researchers have identified eleven or twelve call-types produced by the different species of canids (most of these studies were focused on wild canids) and these have been subdivided into approach-eliciting sounds and withdrawal-elicitng sounds [22], [23], [24], [25], [26]. Yin and McCowan [25] have shown that dog barks are graded vocalizations that range from harsh, low-frequency calls to harmonically rich, higher frequency calls, and that they can be divided into subtypes (disturbance, isolation, and play) based on context, even within individual dogs. Disturbance barks are harsh, low-pitched barks with little amplitude modulation and little pitch modulation. Isolation and play barks on the other hand are more tonal, higher-frequency calls with more modulation in both pitch and amplitude.

The aim of our research was to examine lateralization in the domestic dog and its association with behavioural response to acoustic stimuli by presenting dogs with playbacks of a thunderstorm and of their species-typical vocalizations in order to determine which hemisphere is used to process these sounds and the emotional reactivity expressed. Furthermore we investigated the correlation between laterality of the head orienting response to acoustic stimuli and paw preference to establish whether lateralization of the dog brain occurs on at least two levels of neural organization: sensory and motor.

## Materials and Methods

### Subjects

Subjects were 14 domestic dogs of various breeds (5 Rhodesian Ridgebacks, 2 Boxers, 2 Labrador Retrievers, 2 Border Collies, 1 Dachshund and 2 mixed-breed dogs). Dogs ranged from 2 to 13 years of age (5,6±1,03; mean years±s.e.m.). All dogs (8 females, and 6 males) were pets living in households. Only one male and one female were entire. No subject had been tested previously.

### Sound recording

Recording of vocalizations for playback were made using a directional microphone (Senheisser ME-66+K6) and a digital recorder (Marantz PMD-670) at a 16-bit quantization and 44.1 kHz sampling rate. Vocalizations were filtered and edited using sound software (Audition 2.0, Adobe Inc.). The recorded samples were of three kinds of vocalization, categorized according to the work of Yin and McCowan [25]: (1) a disturbance situation in which a stranger knocked on the door of the owner's house, (2) an isolation situation in which the dog was in a room of the house isolated from its owner and (3) a play situation in which either two dogs or a human and a dog played together. The sound of a thunderstorm was taken from commercial CD (“Loud noises, to calm your dog”, Sound Design Studios, 2000). Three samples of each type of sound were collected, and each sample used in playback lasted for 10 seconds and contained 3 seconds of sound (vocalization or thunderstorm) followed by 7 seconds of silence.

### Sound playback and Head-orienting response

**Figure 1:**
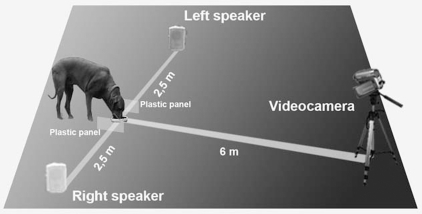
Schematic representation of the testing apparatus.

A digital portable player (Mpio FL 70®) and two speakers (JBL N24AWII®) connected to an amplifier (Yamaha RX-N600®) were used to play sound samples back in the owner's back yard. Each dog was tested with its favourite dry dog pellets in a bowl. The two speakers were placed 2.5 m to the right and left side of the feeding bowl; the speakers and bowl were all in a straight line (see Fig. 1). Two plastic panels (30 cm high, 50 cm in depth) were located on the two sides of the bowl to centre the position of the dog with respect to the speakers and the video recording area during the experiment (Figure 1). Once the dog had commenced feeding, a sound was played at the same time from both speakers. The different sounds were played in random order from the two speakers and the side on which each speaker was placed was alternated. The sounds, were played for 3 seconds at a volume of 60–80 db at the distance of the dog's head from the speakers (measured with a Precision Sound Level Meter, Type 2206, Brüel & Kjær,Nærum, Denmark at 2.5 m from the speakers in a soundproof room) and there was a 1-min interval between each presentation, provided that the dog remained at the food dish. The playback was stopped if the dog stopped feeding. Each dog was tested during a single session of one hour at weekly intervals until a set of 10 playbacks of each sound was achieved. The tester recorded the dog's head-orienting response to the speakers in response to the playbacks using a digital videocamera placed in front of the bowl at a distance of 6 m. Three responses were possible: turn right, turn left and no response if the dog did not turn the head within 5 seconds of playing the sound. The time to resume feeding from the bowl after playbacks was also measured (5 minutes was considered the maximum time allowed to resume feeding).

In the binaural auditory test that we have used it is assumed that, if the subject turns toward the speaker on its right side, the acoustic input is processed primarily by the left hemisphere, at least for the initial attention to the stimulus, and vice versa if it turns toward the left side [27]. The direction of the head turn, which is an unconditioned response, is therefore considered to be an indicator of a contralateral hemispheric advantage in attention to the auditory stimulus [12].

### Behavioural score

The behavior of the dogs was video recorded continuously during sessions and up to 5 minutes after a session in which the dog did not return to the food dish. The video footage was subsequently used to score any of the following listed behaviour: vocalization, barking, whining, panting, salivating, ears back, shaking of the body, urinating, defecating, tail between the legs, running away, hiding, seeking attention from the tester, lowering of the body posture and freezing. Each performed behaviour was allocated a score of 1, and the total for each dog was used to generate a reactivity index. The highest possible score was 15, and the lowest score was 0.

### Paw preference test

Paw preference was estimated according to the method of Branson and Rogers [8]. Each dog was visited at its owner's home and presented with a Large Classic Kong. The Kong was filled with a mixture of palatable food (meat and dry dog food) and presented to the dog on a flat surface in the backyard of the owner's house. The dog's use of the left (L) or right (R) forepaw or both forepaws together (B) to hold the Kong while eating its contents was recorded until a total of 50 L plus R scores had been collected for each dog irrespective of the number of bimanual scores.

Experiments were conducted in accordance with the *Australian Code of Practice for the Care and Use of Animals for Scientific Purposes* (National Health and Medical Research Council, 1997) and were approved by the University of New England Animal Ethics Committee.

### Statistical analysis

#### Head-orienting response

A laterality index (LI) for the head-orienting response of each dog to playbacks of the different sounds was also calculated using the formula LI = (L−R/L+R), where L and R signify respectively the number of Left and Right head-orienting responses; hence a score of 1.0 represents exclusive head turning to the left side and −1.0 exclusive head turning to the right side. A LI score of 0 indicates equal numbers of turns of the head with the right and the left ear leading. One sample T test was used to detect if the LI was different from 0.

#### Paw preference

The first 50 L or R paw scores were used to calculate a binomial z score for each dog to determine whether the paw preference differed significantly from chance. The formula used to calculate this was z = (R−0.5N)/√(0.25N), where R signifies the number of R paw uses and N signifies the sum of L plus R paw uses. Dogs with a positive z score value equal to or greater than 1.96 were R-pawed, those with a negative z score value equal to or less than −1.96 were L-pawed, and the remainder were ambilateral, A (showing no paw preference). A handedness index (HI) was also calculated for each dog (L−R/L+R); hence a score of 1.0 represents exclusive use of the L paw and −1.0 exclusive use of the R paw. The absolute value of HI is the strength of paw preference with the highest possible value of 1.0 indicating the exclusive use of either the L or R paw. A HI score of 0 indicates equal use of the L and R paws.

For all statistical tests, SPSS software was used, and the results were considered significant if p<0.05.

## Results

### Head-orienting response

**Figure 2:**
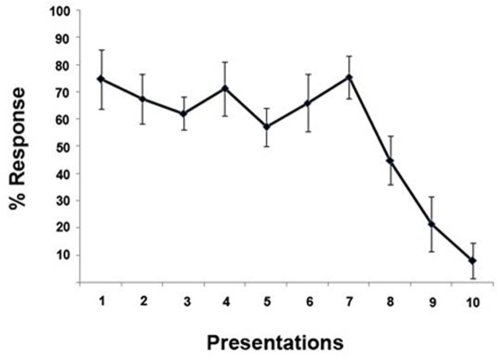
% Response index (%Res). %Res for the head-orienting response of each dog to playbacks calculated for all of the 10 presentations of the different sounds.

First, a % Response index (%Res) for the head-orienting response of each dog to playbacks was calculated for all of the 10 presentations of the different sounds, using the formula %Res = (L+R/L+R+N), where L and R signify respectively the number of Left and Right head-orienting responses, and N signifies “No response” (i.e. if the dog did not turn its head within 5 seconds after presentation of the sound). The %Res revealed that no subjects habituated to the acoustic stimuli during the first seven presentations but a pronounced decrease in the %Res was observed in the last three presentations (see Figure 2). All the data were subsequently analysed using only the first seven presentations. The data for percentage of response were analysed to see whether the stimuli differed in terms of eliciting a head orienting response. A GLM analysis for repeated measures of the data for %Res revealed that there was no difference between sounds on this measure (“disturbance”, “isolation”, “play” and “thunderstorm”) (F(3, 39) = 0.127, p = 0.944).

**Figure 3:**
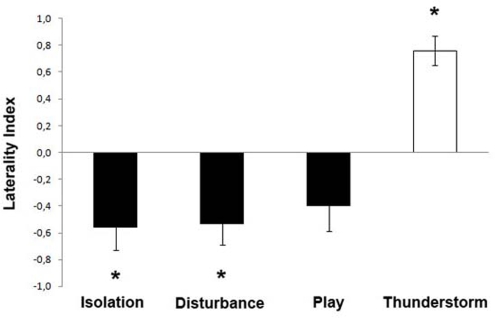
Laterality index (LI). LI for the head-orienting response of each dog to playbacks over the first 7 presentations: a score of 1.0 represents exclusive head turning to the left side and −1.0 exclusive head turning to the right side; * = P<0.01 (two-tailed t-tests).

Regarding the Laterality Index determined for the head-orienting response, a significant main effect of stimulus was observed (F(3,39) = 22.954, p = 0.000): post-hoc analysis (Fisher's Protected LSD) revealed that this main effect of stimulus was due to the response to the “thunderstorm” sounds being different from the responses to all other sounds (P<0.01 for all comparison between thunderstorm and the other stimuli), as can be seen from Figure 3. For Isolation and Disturbance call types, subjects consistently turned their head to the right side (Isolation call: t(13) = −3.172, P = 0.007; Disturbance call: t(13) = −3.238, P = 0.006, two-tailed t-tests) and, although there was a trend for the same side orienting bias for the Play call (t(13) = −2.048, P = 0.061, two-tailed t-tests), this was not significant. Nevertheless, as shown in Figure 3, dogs showed a comparable head orienting response for all three call types. In contrast, a significant head orienting response to the left side was found when dogs attended to playbacks of “thunderstorm” (t(13) = 6.505, P = 0.000, two-tailed t-tests).

### Latency to resume feeding

**Figure 4:**
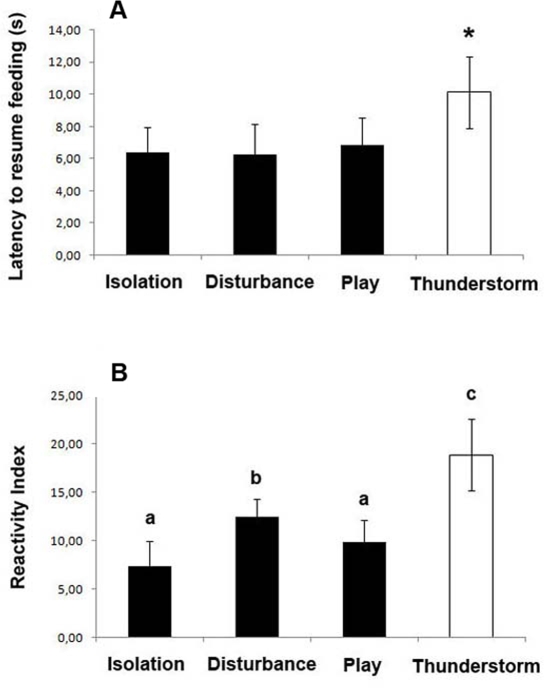
Latency to resume feeding and Reactivity Index (RI). A, The mean (and 95% confidence interval) of latency to resume feeding from the bowl for each dog for each stimulus over the first 7 presentations (5 minutes was considered the maximum time to resume feeding); * = P<0.05. B, Data for the mean (and 95% confidence interval) score of the Reactivity Index determined from the Behavioural score for each dog for each stimulus over the first 7 presentations; (a,b) =  P<0.05; (a,c), (b,c) =  P<0.01.

A significant main effect of acoustic stimuli was also identified in mean latency to resume feeding (F(3,39) = 2.883, p = 0.048): post-hoc analysis (Fisher's Protected LSD) revealed that the latency was longer for “thunderstorm” than for any other sound (P<0.05 all comparisons between thunderstorm and the other stimuli) (see Fig. 4A). In addition the dogs were less likely to resume feeding from the bowl within the testing session if they turned left than if they turned right and this was the case irrespective of the sound presented: feeding was not resumed within 5 minutes in 44 occasions and 42 of these were left turns and only 2 were right turns. Even when feeding was resumed within the testing session, left turns were followed by significantly longer latencies (10.38±2.19 s) than right turns (4.09±0.51), irrespective of the stimulus (t(13) = 3.204, p = 0.007).

**Figure 5:**
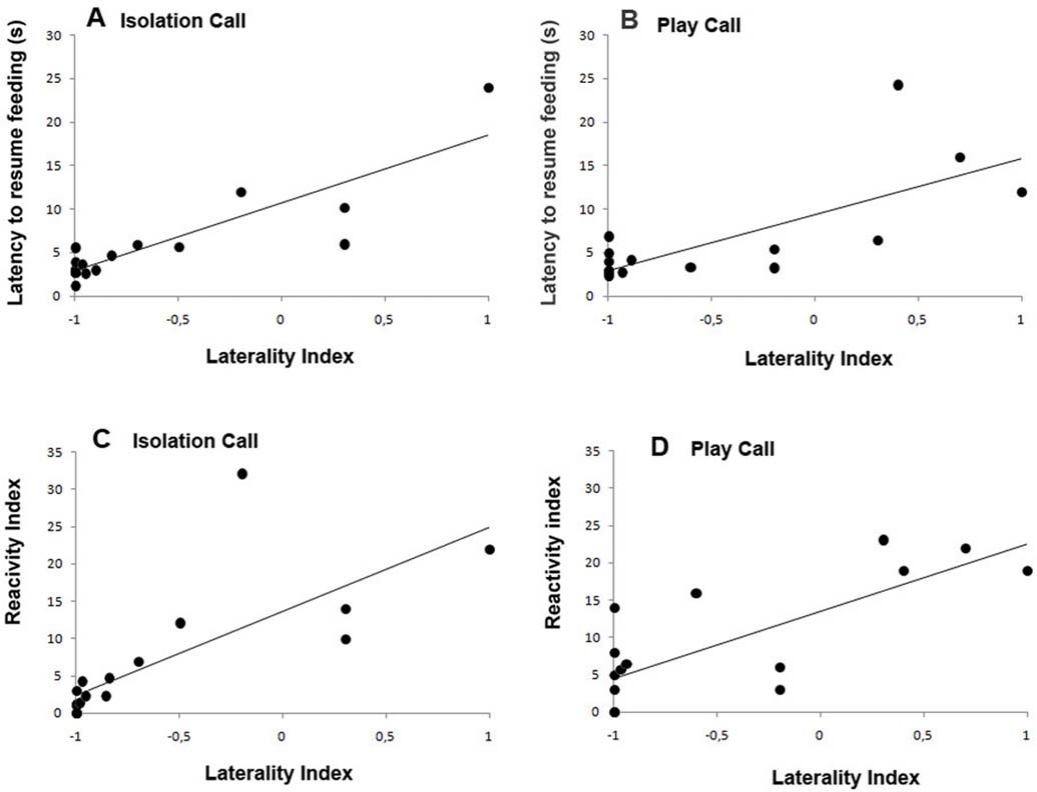
Data for the significant correlations discussed in the text between Laterality Index and the Latency to resume feeding (A, Isolation; B, Play) and between Laterality Index and the Reactivity Index (C, Isolation; D, Play); Data presented are means calculated for each dog over the first 7 presentations.

A Pearson's correlation comparing the Laterality Index and the Latency to resume feeding demonstrated a strong positive and significant association for the three calls: “isolation” (r(12) = 0.873, P = 0.000); “play” (r(12) = 0.734, P = 0.003); “disturbance” (r(12) = 0.640, P = 0.014) Figure 5 (A, B).

### Behavioural score

Regarding the behavioural score, a Reactivity Index was determined by calculating the total number of manifested behaviours (each manifested response was allocated a score of 1) for each dog and for each stimulus over the first 7 presentations. A GLM analysis for repeated measures of the data for Reactivity Index (RI) revealed that there was a significant difference between acoustic stimuli (“disturbance”, “isolation”, “play” and “thunderstorm”) (F(3,39) = 10.431, p = 0.000): post-hoc analysis (Fisher's Protected LSD) revealed that dogs were more reactive when they attended to playbacks of “thunderstorm” (P<0.05 in all measures), and that there was also a significant difference between the RI of the “disturbance” call (m = 12,42±1,91) and the RI of the “play” call (m = 9,85±2,30) (P = 0.016), and between the RI of the “disturbance” call and the RI of the “isolation call” (m = 7,35±2,62) (P = 0.018) but not between the “isolation” and the “play” call (P = 0.115) (see Figure 4B).

The LI in the head orienting response to calls was also positively and strongly correlated to the RI for calls: “isolation” (r(12) = 0.755, P = 0.002) Fig. 5C; “play” (r(12) = 0.764, P = 0.001) Fig. 5D; “disturbance” (r(12) = 0.717, P = 0.004).

**Figure 6:**
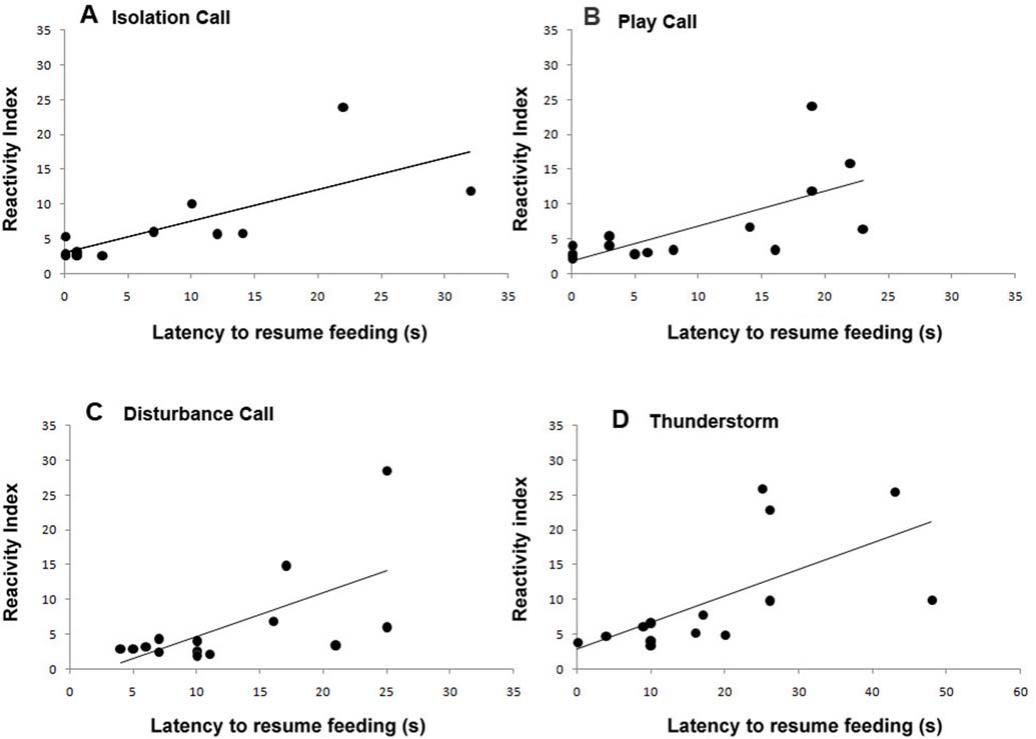
Data for the significant correlations discussed in the text between Reactivity Index and the Latency to resume feeding for all acoustic stimuli (A–D); Data presented are means calculated for each dog over the first 7 presentations.

Finally the Latency to resume feeding was correlated with scores for the RI and a significant positive correlation was found among all acoustic stimuli: “isolation” (r(12) = 0.760, P = 0.002); “play” (r(12) = 0.678, P = 0.008); “disturbance” (r(12) = 0.632, P = 0.015); “thunderstorm” (r(12) = 0.637, P = 0.014) Fig. 6(A–D).

### Paw Preference

The z-score calculations of data collected on the 14 dogs tested on the Kong test identified 6 dogs as being significantly L-pawed, 5 as significantly R-pawed, and 3 as ambilateral. The association between paw preference (L, R, A) and LI in the head orienting response to all acoustic stimuli was analyzed using GLM. Paw preference was found to have no significant effect on the LI data (F(6, 33) = 0.389, p = 0.881). GLM analysis revealed also that there was no significant effect of paw preference on Latency to resume feeding (F(6, 33) = 0.188, p = 0.978) and on Reactivity Index (F(6, 33) = 0.234, p = 0.962).

### Sex Ratio

A GLM analysis for repeated measures revealed that there was no significant effect of sex on the LI in the head orienting response (F(6, 33) = 0.900, p = 0.451), Latency to resume feeding (F(6, 33) = 1.407, p = 0.257) or Reactivity Index (F(6, 33) = 0.648, p = 0.589) for any of the acoustic stimuli.

## Discussion

We found that dogs turned their head to the right side (left hemisphere) in response to conspecific vocalizations, but to the left side in response to the sound of the thunderstorm. This finding is consistent with other results obtained for rhesus monkeys (*Macaca mulatta*), which showed that adult subjects turned with the right ear leading (left hemisphere) in response to presentation of conspecific vocalizations (12 call types that could be separated into three broad categories: aggressive, fearful, and affiliative), but turned with the left ear leading (right hemisphere) in response to the alarm call of a seabird (*Arenaria intepres*) [10]. Psychophysical experiments also indicate that Japanese macaques (*Macaca fuscata*) exhibit a right ear/left hemisphere bias for discriminating between two types of affiliative vocalizations from their repertoire, providing evidence for the existence of a left hemisphere bias for processing conspecific vocalizations [27].

Hauser and colleagues [27] also investigated whether experimental manipulations of the conspecific calls of rhesus macaques beyond the species-typical range of signal variation would cause a change in perceptual asymmetry, either reversing the pattern (right to left ear) or wiping it out (no asymmetry). Results showed that for some call types within the repertoire (an affilitaive signal “grunt” and an alarm signal “shrill bark”) temporal manipulations of interpulse interval outside the range of natural variation either eliminated the orienting bias or caused a shift from right- to left-ear bias. In parallel with the manipulation of interpulse interval Ghazanfar and colleagues [28] showed that rhesus macaques switched from right to a left ear-orienting bias for both harmonic arches and shrill barks played backwards.

In humans, it has been suggested that asymmetrical processing of complex sounds as in speech does not depend on semantic, but rather on acoustic stimulus characteristics [29], [30]. We cannot entirely rule out the possibility that, in dogs, it is the acoustic features that determine the direction of turning but our method of repeated presentations of the same stimuli to individual dogs allows us to assess unusual occasions when dogs happened to break with their typical pattern and turn to the left in response to hearing a vocalization. In these cases the reactivity of the dogs was elevated, which shows that the side of turning is associated with the processing and response and seems not to be determined by the acoustic features of the stimulus per se. Moreover, two of the dogs we tested were extremely fearful and they consistently turned to the left in response to hearing the vocalizations. In other words, processing is different in each hemisphere and the lateral bias we determined matches the subsequent behaviour of the dogs. Further research is needed to determine the responses of dogs to temporal manipulations of their vocalizations.

Results from all of the experiments described above are consistent with our results and with the general interpretation that the left hemisphere is specialized to process conspecific vocalizations and familiar stimuli. It is interesting to note that in our experiment the trend to turn the head to right side in response to presentation of “play” calls was not significant: in a recent work Molnar et al. [26] reported the results of the first analysis and classification of companion dog barks using machine learning algorithms. The algorithm's task was to learn which acoustic features of the barks, which were recorded in different contexts and from different individuals, could be distinguished from another vocalization. Results showed that poorest recognition rate was achieved for the barks recorded in the “play” contexts. This result parallels our finding: the “play” call is a less distinguishable vocalization than the “isolation” and “disturbance” call and this phenomenon could explain the weaker bias for the “play” call in the head orienting response provided that the dogs sometimes interpreted the call as being outside the normal range (i.e. as in Hauser's experiment) [27].

The left side turning (right hemisphere) bias in the head orienting response to playbacks of thunderstorms is consistent with the interpretation that the right hemisphere is more active in processing sounds falling outside the species-typical repertoire but which may be meaningful in terms of particularly salient environmental events [3]. An observation that supports this hypothesis is the left ear (right hemisphere) bias in the rhesus macaques seen in response to the turnstone's alarm call in the experiment by Hauser and Andersson [10] (see above). This call contains meaningful information for rhesus macaques that may be used to predict the presence of humans and the consequent delivery of monkey chow or attempts to trap the monkeys for biomedical purposes. In dogs, the sound of the thunderstorm could be an event that may be useful in predicting a change in the owner's behavior (some people are anxious during a storm) and of the environment (switching off the light, closing doors and windows) both of which can modify the dog-owner interactions. Testing dogs using an auditory evoked response (AER) technique, Adams et al. [21] found that right-hemisphere activity reliably discriminates voicing contrasts along boundaries important for human phonetic contrast: the perception of voicing contrasts of human speech could represent a clear example of how the right hemisphere analyses sounds which contain meaningful information for dogs (the owner's voice) but which fall outside the canine species-typical repertoire.

Alternatively, the sound of the thunderstorm could increase the arousal state of the dog and hence activity of the right hemisphere, which has been associated with the expression of intense emotions, such as aggression, escape behavior and fear (summarized in Rogers) [31]. In dogs, this hypothesis is confirmed by a recent work of Quaranta et al. [6], which showed that right brain activation (higher amplitude of tail wagging movements to the left side of the dog's body) occurs when the animal views stimuli that could be expected to elicit fear and withdrawal tendencies, such as a dominant unfamiliar dog.

Moreover, in our experiment, when dogs turned left (right hemisphere), regardless of the sound presented, they were less likely to resume feeding from the bowl within the testing session than if they turned right and, even when feeding was resumed within the testing session, it was after a longer latency following a left turn than following a right turn. In other words, activation of the right hemisphere led to a longer latency to resume feeding. The latency to resume feeding is an indirect behavioral parameter that has been interpreted as an indicator of fear in several animal models [32], [33]. Further evidence comes from the positive correlation between the left-side turning bias (activation of the right hemisphere) in the head orienting response and the latency to resume feeding from the bowl for the three vocalizations (isolation, disturbance and play). This result is evidence that conspecific vocalizations are not always processed by the left hemisphere, as Hauser found in monkeys [10], since the right hemisphere is used for processing vocalizations when they elicit intense emotion, including fear. Additionally we found among all calls a positive correlation between the left turning bias in the head orienting response (right hemisphere activation) and the behavioural score of reactivity, which is a direct index used to express the fear and emotional state of the dog [8], [34].

Regarding paw preference, no association was found between the use of the paws to handle a Kong and the LI in the head orienting response. A similar result has been observed in a recent research on a non-human primate (the gray mouse lemur), which showed that asymmetries in communication sound perception are not related to hand preference [12]; although these authors observed that only males exhibited significant orientation asymmetries in communication sound perception, we did not find any sex difference in our experiment.

Overall, results from our experiments have revealed that dogs usually process their species-typical vocalizations using the left hemisphere and the thunderstorm playbacks using the right hemisphere. This result is consistent with the different specializations of the right (analysis of novelty/fear) and the left (analysis of familiar stimuli) hemispheres reported previously. The right hemisphere is used for processing vocalizations on occasions when they elicit intense emotion, including fear. For processing conspecific calls such as those signalling isolation, play or disturbance we found a positive correlation between the use of the right hemisphere and the emotional state of the animal. Nevertheless, dogs usually use the left hemisphere to attend to their species-specific vocalizations and this adds further evidence to the evolutionary continuity of the left hemisphere's involvement in vocal communication.
